# The effect of SENATOR (Software ENgine for the Assessment and optimisation of drug and non-drug Therapy in Older peRsons) on incident adverse drug reactions (ADRs) in an older hospital cohort – Trial Protocol

**DOI:** 10.1186/s12877-019-1047-9

**Published:** 2019-02-13

**Authors:** Amanda H. Lavan, Denis O’Mahony, Paul Gallagher, Richard Fordham, Evelyn Flanagan, Darren Dahly, Stephen Byrne, Mirko Petrovic, Adalsteinn Gudmundsson, Olafur Samuelsson, Antonio Cherubini, Alfonso J. Cruz-Jentoft, Roy L. Soiza, Joseph A. Eustace

**Affiliations:** 1 Department of Medicine (Geriatrics), University College Cork, Cork University Hospital, Cork, Ireland; 2 Pharmaceutical Care Research Group, School of Pharmacy, University College Cork, Cork, Ireland; 3 Health Research Board Clinical Research Facility-Cork, University College Cork, Cork University Hospital, Wilton, Cork, Ireland T12 DC4A; 4 Vakgroep Inwendige Ziekten (Geriatrie), Universiteit Gent, UGent, Ghent, Belgium; 5 Landspitali University Hospital Reykjavik, Reykjavik, Iceland; 6 Geriatria, Accettazione geriatrica e Centro di ricerca per l’invecchiamento, IRCCS INRCA, Ancona, Italy; 7 Hospital Universitario Ramón y Cajal (IRYCIS), Madrid, Spain; 8 NHS Grampian and University of Aberdeen, Aberdeen, Scotland; 9 Health Economics, University of East Anglia Medical School, Norwich, England

**Keywords:** Adverse drug reactions, Randomized controlled trial, Older adults, Hospitalization, Computer software, Intervention study, Medication alert systems, Polypharmacy, Multimorbidity

## Abstract

**Background:**

The aim of this trial is to evaluate the effect of SENATOR software on incident, adverse drug reactions (ADRs) in older, multimorbid, hospitalized patients. The SENATOR software produces a report designed to optimize older patients’ current prescriptions by applying the published STOPP and START criteria, highlighting drug-drug and drug-disease interactions and providing non-pharmacological recommendations aimed at reducing the risk of incident delirium.

**Methods:**

We will conduct a multinational, pragmatic, parallel arm Prospective Randomized Open-label, Blinded Endpoint (PROBE) controlled trial. Patients with acute illnesses are screened for recruitment within 48 h of arrival to hospital and enrolled if they meet the relevant entry criteria. Participants’ medical history, current prescriptions, select laboratory tests, electrocardiogram, cognitive status and functional status are collected and entered into a dedicated trial database. Patients are individually randomized with equal allocation ratio. Randomization is stratified by site and medical versus surgical admission, and uses random block sizes. Patients randomized to either arm receive standard routine pharmaceutical clinical care as it exists in each site. Additionally, in the intervention arm an individualized SENATOR-generated medication advice report based on the participant’s clinical and medication data is placed in their medical record and a senior medical staff member is requested to review it and adopt any of its recommendations that they judge appropriate. The trial’s primary outcome is the proportion of patients experiencing at least one adjudicated probable or certain, non-trivial ADR, during the index hospitalization, assessed at 14 days post-randomization or at index hospital discharge if it occurs earlier. Potential ADRs are identified retrospectively by the site researchers who complete a Potential Endpoint Form (one per type of event) that is adjudicated by a blinded, expert committee. All occurrences of 12 pre-specified events, which represent the majority of ADRs, are reported to the committee along with other suspected ADRs. Participants are followed up 12 (+/− 4) weeks post-index hospital discharge to assess medication quality and healthcare utilization.

This is the first clinical trial to examine the effectiveness of a software intervention on incident ADRs and associated healthcare costs during hospitalization in older people with multi-morbidity and polypharmacy.

**Trial registration number:**

Clinicaltrials.gov NCT02097654, 27 March 2014.

## Background

Adverse drug reactions (ADRs) represent a significant and growing global healthcare problem, especially in the elderly population. ADRs may both cause hospitalization, and/or occur in hospital. Approximately 11% of hospital admissions in adults aged ≥65 years are caused by ADRs [1], while one in ten hospitalised patients are reported to experience an ADR during their inpatient stay [2]. Older age [2], female gender [3], multi-morbidity [4], polypharmacy [4] and frailty [5] are significant predictors of ADRs and are central to the GerontoNet ADR prediction tool described in 2010 [6]. Since approximately half of all ADRs are thought to be preventable [7], they represent a major, modifiable contributor to morbidity and mortality in older subjects.

Inappropriate prescribing and excessive polypharmacy are both linked to ADRs, but few interventions addressing these risks have been evaluated. A recent single centre clinical trial showed that the application of the ‘Screening Tool of Older Persons’ Prescriptions’ (STOPP) and ‘Screening Tool to Alert doctors to the Right Treatment’ (START) screening tools by an experienced geriatrician within 48 h of admission reduced incident in-hospital ADRs from 21.0 to 11.7% [8]. Patients recruited to this study were adults 65 years and older admitted acutely to hospital; patients were excluded if they were admitted directly to the intensive care unit, under geriatric medicine, old age psychiatry, clinical pharmacology or palliative medicine. Additional studies have shown that the manual (i.e. not delivered by software) application of STOPP/START criteria improves medication appropriateness [9, 10] and reduces the risk of falls [11]. However, these trials have been single centre and unblinded. Furthermore, the lack of sufficient geriatric medicine specialists limits the applicability of this manual approach. To overcome these limitations, a novel software tool called SENATOR (Software ENgine for the Assessment and optimisation of drug and non-drug Therapy in Older peRsons) was developed, which automatically provides individualized STOPP/START and other relevant recommendations based on standardized inputs. Given the support in the literature for the efficacy of the STOPP/START recommendations, we decided to conduct a pragmatic trial to quantify the effectiveness of providing the individualised SENATOR software report to the participating patient’s attending hospital clinician for review and implementation as he/she judges appropriate compared to usual clinical care, with the aim of reducing in-hospital ADRs amongst older adults in a real world setting across 6 European hospitals.

## Methods

The SENATOR Trial is a multinational, pragmatic, parallel arm Prospective Randomized Open-label, Blinded Endpoint (PROBE) controlled trial [12]. It is pragmatic in nature, in that it aims to evaluate the SENATOR software in real world clinical practice with decisions being made by busy clinicians. The trial is funded by the European Union Framework Programme 7 and includes a diverse geographical make-up, with participation of six large university-affiliated hospitals from across Europe (Ireland, Scotland, Iceland, Spain, Italy and Belgium). The trial is registered with clinicaltrials.gov (NCT02097654).

### Entry criteria

To be eligible for inclusion, participants have to be 65 years or older and must be admitted to a hospital in a non-elective manner, under the care of a medical or surgical service, and have an initial management plan in place (Table 1). Subjects are required to have 3 or more active co-morbidities, defined as conditions requiring ongoing medical therapy. Patients are excluded if they are admitted under the care of specialists in geriatric medicine or clinical pharmacology, or if they have already been reviewed by these services or are scheduled to undergo such a consultation, since these services provide similar advice to the SENATOR software, potentially supressing the observed ADR event rates and making it more difficult to detect any benefit from the intervention. Similarly, patients admitted to intensive care units or under the care of oncologists, as well as patients with liver failure, renal failure receiving dialysis, and patients with solid organ transplants, will also be excluded, since detailed specialist scrutiny and adjustment of prescribed medications occur regularly in these settings. As ADR rates are highest in the initial days following admission, patients are required to give their informed consent within 48 h of arrival to hospital and be randomized within 60 h of arrival. To allow for adequate follow-up, subjects are excluded if the anticipated length of their hospital stay is less than 48 h, if there is an existing documented intention to transfer them to another hospital, or if the attending clinician believes the subject’s life expectancy is less than 3 months.

**Table 1:** SENATOR Trial: Inclusion and exclusion criteria

Inclusion criteria	
• ≥ 65 years • Admitted with an acute illness under the care of a specialist other than a geriatrician OR clinical pharmacologist OR palliative care physician OR oncologist OR haematologist • Consented into the study ≤60 h from time of arrival to the hospital • Anticipated in hospital stay of > 48 h, in the opinion of the treating physician • ≥ 3 active (requiring current medication) chronic medical disorders	
Exclusion criteria	
• Elective hospitalisation • Patients actively participating in another clinical trial of a medicinal product • Documented plan for consultation with Geriatric Medicine, Clinical Pharmacology, Palliative Medicine, Clinical Oncology or Haematology specialist services at the time of study recruitment • Admission directly to an intensive care unit • Primary acute psychiatric illness (excluding delirium) • Admission with non-accidental overdose/self-harm • Patients still under the care and responsibility of Emergency Department Physicians • Patients considered by the attending clinician to have a life expectancy of < 3 months • Anticipated immediate transfer to alternative non-participating clinical service/hospital • Receiving renal dialysis • Clinical diagnosis of acute liver failure • Solid organ transplant recipient • Admitted to hospital > 60 h at time of attempted randomization.	

### Intervention

The SENATOR software was designed by the project consortium and implemented by the Clanwilliam Group®. It produces a report, in the clinician’s native language, that identifies potential risks, and opportunities for improvement, in the participants’ current medication list. The report has 5 components: [1] recommendations for modifying or discontinuing a current medication; [2] recommendations to initiate a new medication (both based on the published STOPP/START guidelines); [3] identification of major drug-drug and [4] drug-disease interactions (both based on SafeScript®software and other local drug-drug and drug-disease interaction databases) [5] and non-pharmacological recommendations considered complementary to patients’ drug therapy. STOPP/START is a widely used series of heuristic rules aimed at optimizing drug prescribing in older patients [13], which has been validated in a range of settings. SafeScript® is a validated software system which uses the Summary of Product Characteristics (SPCs) of ATC coded medications in conjunction with ICD-10 coded conditions that was developed in the UK [14]. The non-pharmacological recommendations in the SENATOR report were based on the ‘**O**ptimal evidence-based **N**on-drug **T**herapies in **O**lder **P**eople’ (ONTOP) programme [15]; these evidence-based recommendations were developed independent of the trial as part of the same FP7 funded project. Only the ONTOP recommendations aimed at reducing the occurrence of incident delirium were completed and available for inclusion in the SENATOR report version used within the current trial. Since all older subjects who are hospitalized are at risk for developing delirium, the recommendations are provided to all subjects randomized to the SENATOR intervention, except those who already have delirium at the time of recruitment. The ONTOP recommendations are included by way of a demonstration of the potential utility of SENATOR as a potential mechanism for promoting non-pharmacological therapies i.e. as a proof of concept. However, the study was not powered with the aim of estimating the benefits of the ONTOP component.

In the intervention arm the participant’s clinical team receive an individualized SENATOR report, at a single time point within 60 h of hospital admission. Control arm subjects and their doctors receive no additional study specified intervention. All subjects have usual clinical management whereby as part of routine care clinicians routinely review and adjust medications according to their local practice. In addition, in one site the majority of hospitalized patients undergo a routine review of their medications by a dedicated internal liaison team or by a hospital pharmacist.

As SENATOR is a decision support tool, it efficiently provides in a single report a range of evidence based recommendations, derived from general considerations. Given the multiple complexities of clinical care, none of these recommendations is mandated. Rather clinicians are requested to review them in the context of the patient’s unique clinical circumstances and to make any alterations in accordance with the clinician’s best judgement.

### Randomization

The patients are randomized into one of the two trial arms with a 1:1 allocation ratio. Randomization is stratified by study site and by admitting service type (i.e. medical vs. surgical). The stratum-specific randomization lists are generated using random block sizes by an independent statistician.

### Allocation concealment

Trial allocation is delivered using an interactive Web-Response System developed in conjunction with the main trial database by Clininfo®, the data management partner company within the SENATOR project. Subject allocation is released once all necessary baseline information data have been entered into the trial database and a decision is made to randomize. In subjects randomized to the active intervention, trial data are automatically transferred to the cloud based SENATOR software engine maintained by Clanwilliam Health®, and the resulting SENATOR report is automatically emailed to the local trial research staff at a designated email.

### Report dissemination

The local research team members place a printed copy of the SENATOR report in the medical record or in the case of two sites that exclusively use an electronic patient record, uploads it electronically. The researcher further contacts a member of the clinical team and emails the attending physician alerting him/her of the report’s existence and requesting the attending physician to review it and consider its recommendations. No specific measures are applied to otherwise encourage or oblige the clinician to adopt any of the report’s recommendations. Given the nature of the intervention it is not possible to effectively mask the intervention from the clinical team or from the on-site researchers.

### Trial administrative structures

The trial is coordinated by a dedicated Trial Coordination Committee based in the Health Research Board (HRB) Clinical Research Facility at University College Cork. A Trial Steering Committee, comprising of the SENATOR grant work package leaders, meets regularly by teleconference and supervises progress and communication including protocol amendments. An independent seven member Scientific Advisory Board meet annually and review overall trial progress. The Scientific Advisory Board members are all senior academic clinicians, including five geriatricians, one clinical pharmacologist and one pharmacist. An independent Ethics and Data Safety Committee consisted of an academic geriatrician, a senior hospital internal medicine physician with an interest in ethics, a medical ethicist, and a patient advocate. The committee meets annually to review trial metrics and any potential trial related deleterious events. Trial Deleterious Events are also assessed by the Trial coordinating Committee in real time.

### Trial staff

The Trial Coordinating Committee is staffed by a dedicated Trial Manager, an Endpoint Liaison Officer (both full time) and a Data Manager, Biostatistician, and Trial Monitor (part-time) who are supervised by the Trial Coordinating Investigator. Each of the six sites is led by a site Principal Investigator (PI), who is a senior physician specializing in geriatric medicine and with extensive experience in geriatric pharmacotherapy. These PIs oversee the recruitment, training and conduct of the local trial staff. All site staff are ICH GCP certified researchers with medical, nursing or health science backgrounds. Training consists of live interactive tutorials, online ICD-10 training, case-based ADR adjudications, remote testing of the electronic case report form (eCRF) at each clinical site, a central two day and subsequent one day meeting of all local site staff and web-based site initiation visits prior to recruitment initiation. Audits were performed by the Clinical Research Facility in Cork (CRF-C) monitoring staff who were otherwise independent of the running of the trial.

### Data management

Although pragmatic and using a ‘real world’ approach to the dissemination of the SENATOR report and with a variable degree of engagement by the clinician, it has been necessary to develop and use a trial specific database to gather the inputs used by the SENATOR software in a standardized fashion from the different countries and to collect the trial outcome data which are collected to a far greater degree of precision and accuracy than is usual in clinical practise. To this end, data are entered electronically by the local site research staff into a cloud based database developed by Clininfo®. Data collected is pseudo-anonymized and includes baseline past medical history, current prescription medications and doses, routine blood biochemistry and haematology laboratory data, baseline ECG data, cognitive assessment using the Mini-Mental State Examination [16], functional assessment using the Barthel Index [17], and health related quality of life using the EQ-5D-5 L [18]. At day 14 post randomization or at discharge, whichever occurs first, the EQ-5D-5 L is repeated, the subjects medical conditions reviewed and medications recorded. The case records are reviewed to assess the number of SENATOR recommendations that have been adopted and to identify any potential endpoints as discussed below. At discharge arrangements are made with participants and/or their family for a 12 weeks (+/− 4 weeks) post hospital discharge follow-up call by the local site research staff. This is to enquire about healthcare utilization and any re-hospitalization since the index hospitalization, changes in medications and current health related quality of life.

### Trial endpoints

The Primary Endpoint is the proportion of patients with at least one adjudicated probable or certain, non-trivial incident in-hospital ADR occurring within 14 days of enrollment during the index hospitalization.

### Secondary endpoints include

1. The proportion of patients with at least one adjudicated *possible, probable or certain*, non-trivial incident in-hospital ADR occurring within 14 days of enrollment during index hospitalization.
2. The proportion of patients with at least one adjudicated probable or certain, non-trivial hospital-acquired, *pre-specified (as per* Table 2*) ADR* occurring within 14 days of enrollment during index hospitalization.
3. The *number* of adjudicated probable or certain, non-trivial hospital-acquired ADRs occurring within 14 days of enrollment during the index hospitalization (i.e. the count of Primary Endpoint events).
4. The *number of adjudicated possible, probable or certain*, non-trivial, incident, in-hospital ADR occurring within 14 days of enrollment during index hospitalization.
5. The *number* of adjudicated probable or certain, non-trivial hospital-acquired, *pre-specified (as per* Table 2*)*, non-trivial, incident, in-hospital ADR occurring within 14 days of enrollment during index hospitalization.

**Table 2:** Pre-specified event /ADR for which there is mandatory reporting of all events

Event	Definition
Fall/s	New fall/s
New onset unsteady gait	New onset of unsteady gait that results in poor mobility and unsteady balance
Acute kidney injury	An increase in serum creatinine by 0.3 mg/dl (26.5 μmol/l) within 48 h or an increase in serum creatinine by 1.5 baseline, which is known or presumed to have occurred within the prior 7 days
Symptomatic orthostatic hypotension	A systolic blood pressure drop ≥20 mmHg ± diastolic blood pressure drop ≥10 mmHg within 3 min of standing from the lying or sitting posture associated with symptoms
Major serum electrolyte disturbance	A sodium (Na) of < 130 mmol/l or > 145 mmol/l and/or a potassium (K+) < 3.5 mmol/l or > 5.2 mmol/l and/or a corrected calcium (Ca++) < 2.1 mmol/l or > 2.7 mmol/l
Symptomatic bradycardia	Heart rate of < 50 beats with symptoms
New major constipation	Subjective symptoms of hard stools and/or less than 3 bowel movements per week and/or supported by nursing records
Acute bleeding	Malaena or haematuria or haematemesis or haemoptysis with or without a drop in haemoglobin level > 2 g/dl (not due to rehydration) or associated symptoms (hypotension, tachycardia, pallor) or secondary renal failure
Acute dyspepsia/nausea/vomiting	Subjective symptoms of acute ‘indigestion’/‘upset stomach’ or acute abdominal pain or acute refusal to eat or acute heartburn/acid reflux or acute nausea/vomiting
Acute diarrhoea	New liquid stools reported by the patient or the nursing staff or new liquid stools detected by medical staff on physical examination or new liquid (non-solid) stools occurring more than 3 times in 24 h
Acute delirium	Confirmed by a reliable witness and the DSM-V criteria. Supported by a 4AT ≥ 4 and/or MMSE < 23/30
Symptomatic hypoglycaemia	Symptoms with a blood glucose of < 3.5 mmol/L or < 63 mg/dl.
Unspecified adverse event	For ADEs not specified above e.g. acute liver failure, anaphylaxis

Exploratory Endpoints include all-cause mortality during index hospitalization; re-hospitalisation, composite healthcare utilization, and health related quality of life at 12 weeks post-discharge follow-up; and utilization of non-pharmacological interventions.

### Patient safety

As the SENATOR Trial is not a regulated clinical trial and is not intended for marketing authorization, and in view of the proposed large recruitment of elderly multi-morbid subjects, full pharmacovigilance reporting of all adverse events will not be attempted. Clinicians are encouraged to report any ADRs in accordance with local clinical policies. Any protocol violation, participant injury consequent to study involvement or related to the application of SENATOR recommendations are reported by the site researchers using a standardized ‘Deleterious Event Form’ for review as appropriate by the Ethics Committee(s), Trial Coordinating Centre, Coordinating Investigator and the independent Ethics and Safety Review Group.

### Endpoint ascertainment

Potential ADRs are recorded by the site staff using dedicated primary endpoint assessment forms based on a retrospective review of all the available documentation within the medical record, including medical, nursing and allied health professional case note entries, laboratory values, radiology reports, electrocardiograms and other investigations. The eligible period for endpoint occurrence extends from the day of randomization to the time of index hospital discharge, or until 14 days post randomization, whichever occurs first. In those subjects whose discharge takes place after day 14 the medical record is further reviewed at the time of discharge to determine if any additional evidence from the subsequent hospital course is relevant to the aetiology of a potential study endpoint.

**Fig. 1:**
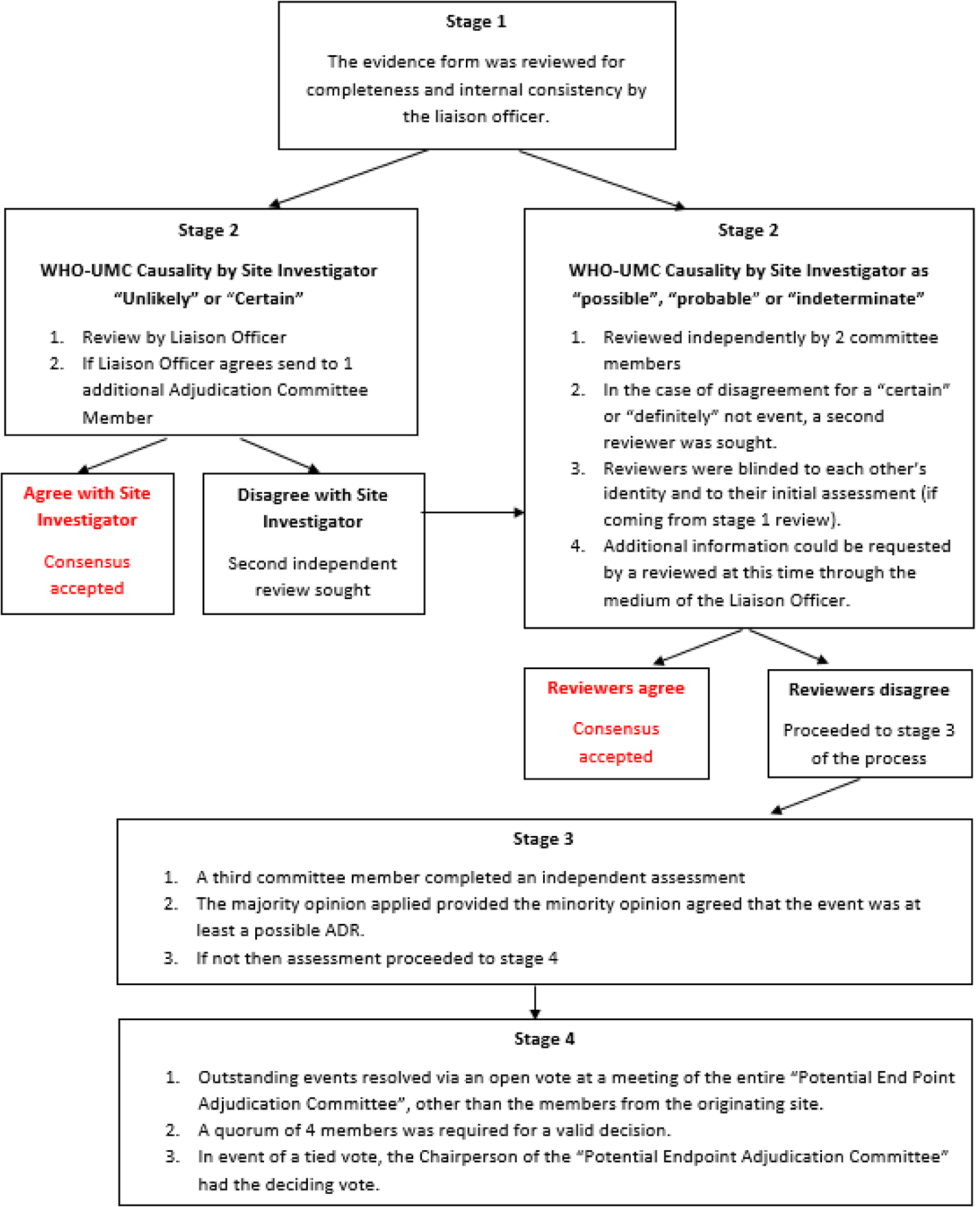
Potential Endpoint Adjudication Process

We identified the 12 most common types of ADRs which represent approximately 80% of all hospital-acquired ADRs in older multimorbid patients (Table 2). For each of these pre-specified types of event, we developed a separate primary endpoint event-specific form, based on a standard template as shown in Fig. 1, to allow for standardised ascertainment of data relevant to that type of event. Any other type of suspected ADRs is recorded using a generic primary endpoint adverse event form. As several medications may be implicated in the same event, the most likely culprit medication, if identifiable, and any other potentially causal or contributory medications implicated in the adverse event are recorded. Generic evidence that supports causality such as temporal relationship, dose response relationship, and response to re-challenge, are recorded, along with any additional qualifying explanatory text used by the local research staff. The unblinded Site Investigator records whether the ADR began prior to randomization (i.e. was prevalent and therefore not part of the primary endpoint) or occurred post-randomization, and his/her own assessment as to the probability of the event being an ADR, and the severity of the event. The strength of the available evidence supporting the event being an ADR is ascertained using the WHO-UMC ADR causality classification system [19], which categorizes ADRs as certain, probable, possible, unlikely or indeterminate. The severity of the event is classified using a modified Hartwig & Siegel scale [20], ranging from trivial (event requires no specific investigation or treatment and has no sequellae) to fatal.

While the recording of evidence supporting an ADR, in a blinded fashion, using the relevant Potential Endpoint Form facilitates the blinded assessment of possible ADRs by the Adjudication Committee, it does not in itself safeguard against the possibility of selective or differential reporting of events to the Adjudication Committee by local site staff. This is especially relevant in the context of an open label trial, as the decision to refer borderline events/possible ADRs is often highly subjective. To avoid against the incomplete or differential submission of potential endpoints according to study arm, we have mandated that all occurrences of the 12 pre-specified events as listed on Table 2 be reported regardless of their presumed aetiology. Compliance with this reporting approach is assessed by on-site monitoring.

Given the difficulty with objectively and reliably recording the number of separate discrete occurrences of a specific type of ADR (e.g. episodes of diarrhoea), especially when using retrospective data review, we have taken the approach of completing a single Potential Endpoint Form for each type of ADR. This summarizes the total available evidence that a particular drug played an etiological role in the genesis of part or the totality of the adverse event that may represent an ADR. Thus, only one form is completed for each type of event regardless of the number of possible discrete occurrences of that particular adverse event during the index hospitalization. A subject could experience several different types of ADRs which are each recorded on their specific case report form or on the generic Potential Endpoint Form, as appropriate.

### ADR adjudication

The Potential Endpoint Form is sent electronically to the blinded members of the Potential Endpoint Adjudication Committee, consisting of the six clinical Site Principal Investigators. Each event is reviewed by up to five Potential Endpoint Adjudication Committee members, excluding members from the same site where the event occurred. Each reviewer independently assesses the likelihood of the event being medication-related and the severity of the event. An algorithm based on the concordance of the independent assessments determines the subsequent number of reviews required to obtain a final decision on whether a non-trivial ADR has occurred or not (Fig. 1). In brief, if the local site Principal Investigator, upon unblinded review of the patient’s case records and the Primary Endpoint Form, grades the event as either ‘unlikely’ or ‘certain’ and the Endpoint Liaison Officer concurs, then the Evidence Form is reviewed by a single blinded Endpoint Committee member (Stage 1). If the stage 1 Reviewer agrees with the Site Principal Investigator, this decision is accepted otherwise the form is reviewed by a second blinded Endpoint Committee member as per Stage 2 below.

**Fig. 2:**
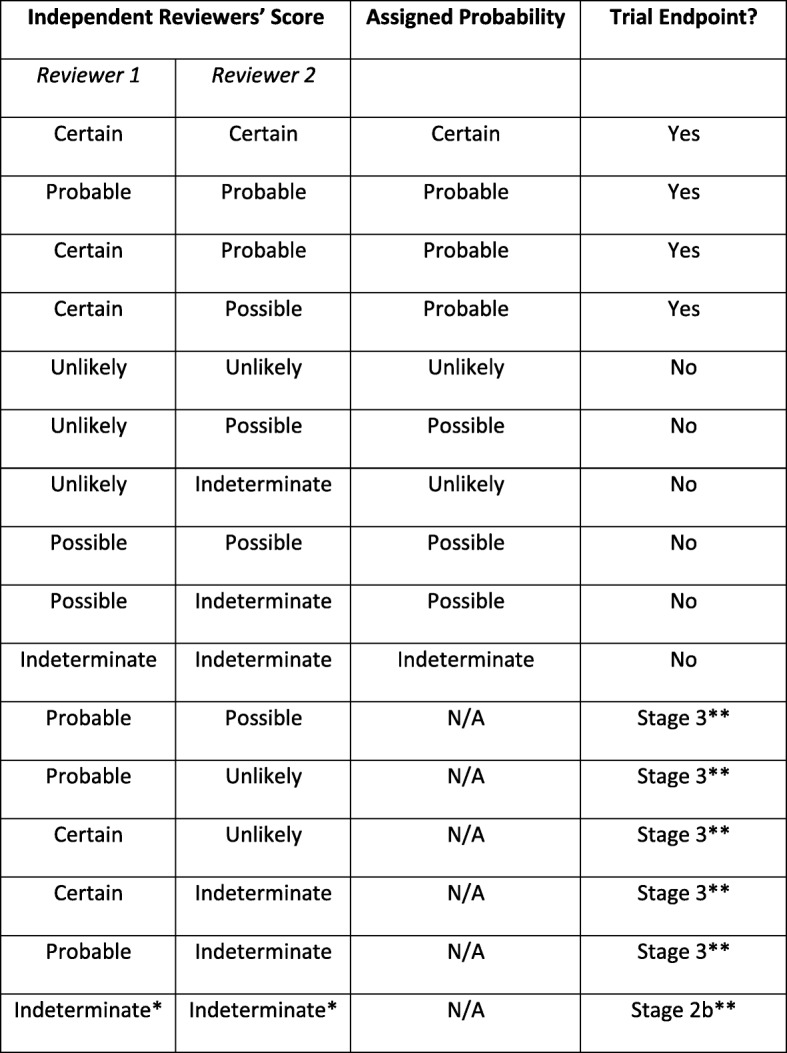
ADR Stage 2 Adjudication Agreement Matrix

All Potential Endpoints judged by the unblinded Site Principal Investigator to be possible, probable, or indeterminate ADRs are directly reviewed independently by two committee members who are blinded to the initial assessment (Stage 2). The adjudicated conclusion is determined by the Stage 2 Agreement Matrix (Fig. 2); where reviewers agree, or any disagreement is minor, an adjudicated result is assigned. For more substantive levels of disagreement, the review progresses to a 3rd blinded Endpoint Committee member (Stage 3) and a majority consensus prevails provided all 3 reviewers judge that the event is at least a possible ADR. Otherwise the event is adjudicated by consensus at a full committee meeting with the Site Principal Investigator being recused.

### Statistical methods

The intention-to-treat study population will consist of all subjects who are randomized. The per-protocol population will consist of those subjects who have at least 60% of the SENATOR report STOPP recommendations acted on by the clinical team. The safety population will include all subjects who provide informed consent.

The primary analysis will compare the odds of subjects in the SENATOR intervention and control groups experiencing the primary endpoint as defined above using a multiple logistic regression in the intention-to-treat population, and adjusting for strata used in the randomization (medical versus surgical admissions and study recruitment site). The null hypothesis is that there is no significant difference between control and SENATOR intervention groups in the adjusted odds of experiencing the primary endpoint. The corresponding estimated effect size will be reported as an odds ratio (SENATOR: control) with 95% confidence intervals. Secondary endpoint analyses will use a similar approach. The null hypothesis will be rejected if this confidence interval excludes the null odds ratio of 1.0. Relevant individual and aggregate data on study related deleterious events will be presented.

### Sample size

**Fig. 3:**
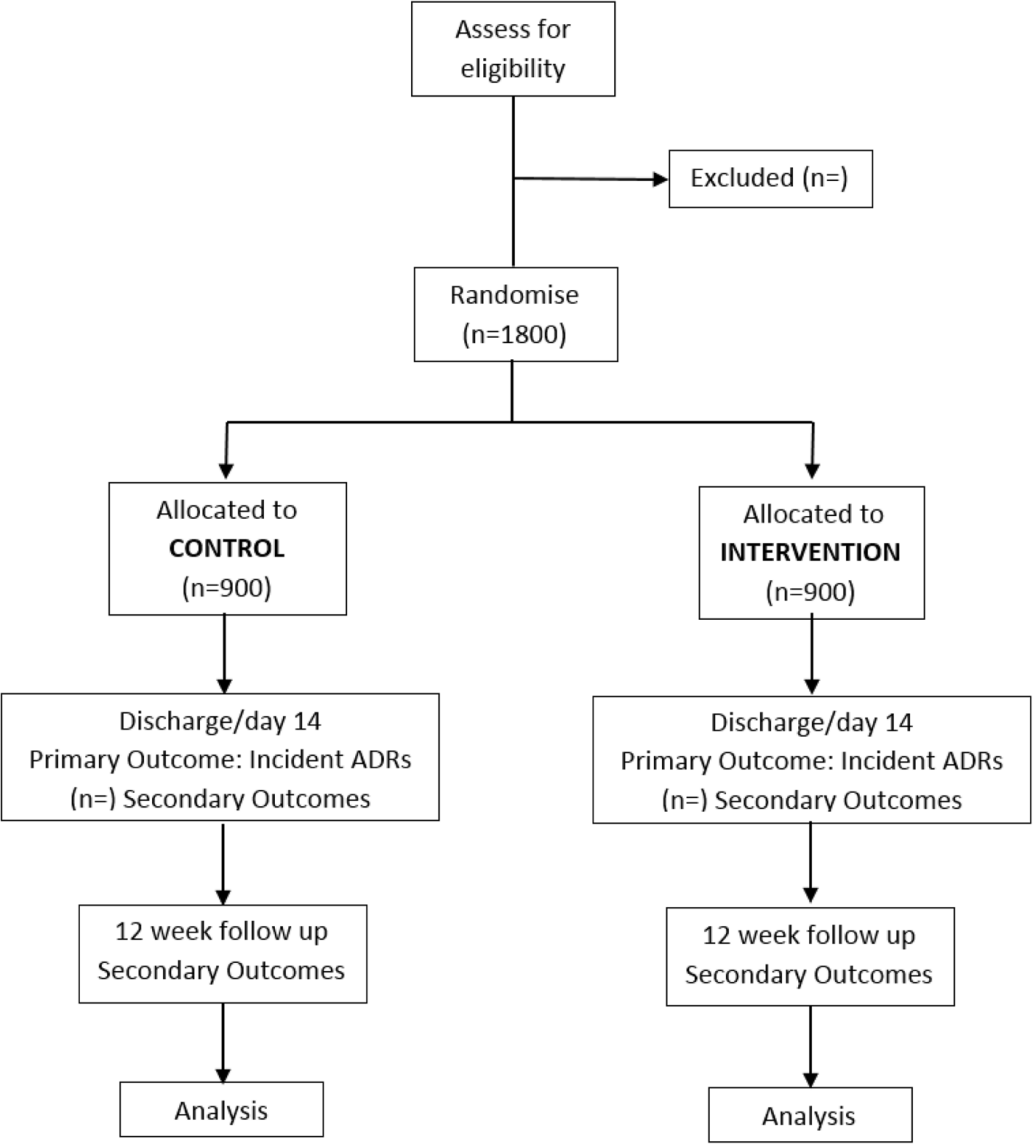
Consort Flow Diagram depicting the SENATOR clinical trial organization

In a recent single blinded trial of the manual application of STOPP/START criteria, conducted at the coordinating investigator site, the observed control group hospital-acquired ADR incidence was 21% compared to an ADR incidence of 11.5% in the intervention group, equivalent to an ADR odds-ratio of 0.45 [8]. This study had similar entry criteria and primary endpoints to the current trial, but differed in that the STOPP/ START recommendations were manually generated and then directly discussed and reviewed with the primary team by a specialist registrar (senior resident) in geriatric medicine. To be conservative, and in view of the more rigorous ADR adjudication processes, we have based our power calculations for the current trial on a lower control group event rate (18%) than that seen in our earlier trial, and we assume a reduced effect size (an odds ratio of 0.65) in order to reflect the potential reduced uptake of recommendations by clinicians. Based on these assumptions, using the method of Farrington and Manning for a trial to test the difference between two binomial events with a two-sided type 1 error rate of 0.05, we achieve 90% power with the proposed recruitment of 900 subjects per trial arm. The planned CONSORT flow diagram for the trial is shown in Fig. 3.

### Economic evaluation plan

The primary economic evaluation will present the cost-effectiveness of the SENATOR intervention and compare the costs with the primary endpoint of the SENATOR intervention against usual care. A cost-utility analysis (CUA) will also be undertaken to estimate the costs and utilities as reported as quality-adjusted life years (QALYs) over the 12-week follow-up period. The incremental cost-effectiveness ratio (ICER) will therefore present the cost per incremental ‘Quality Adjusted Life Year’ of SENATOR in relation to standard management.

Costs for calculation will include medication use, inpatient stay, further hospitalizations, hospital outpatient visits (including physiotherapist, OT), and community-based or health/social care visits. Resource use will be calculated according to the average unit cost of inpatient, outpatient and community visits across sites. In light of the intervention having no clear explicit cost/price, deployment costs of the SENATOR tool from an end-users’ perspective will be estimated by means of a brief user-survey.

The outcomes will be estimated as utilities, as reported from EQ-5D-3 L [18] eCRF data at baseline, day 14 or discharge, and 12-week follow up. The difference in QALY score between the two arms will be estimated using the “area under the curve” method.

The analysis will be undertaken from a payer perspective. Appropriate sensitivity and statistical analysis will be undertaken to test the uncertainty of the effect on the cost and QALY effect.

## Discussion

With a progressive global shortage of specialist geriatricians to treat the rapidly growing frailer multi-morbid older population, pragmatic cost-effective strategies to improve pharmacotherapy for older adults are urgently needed. The majority of drug prescriptions for older multi-morbid people currently and in the future will emanate from clinicians who are neither specialist geriatricians nor clinical pharmacologists. Strategies to optimise the pharmacotherapy of older multi-morbid people and simultaneously minimize drug-related harm must be devised and tested. Previous studies have shown that the implementation of medication reconciliation [21] and comprehensive geriatric assessment [22] reduce inappropriate prescribing and potentially ADRs, but both these approaches are time consuming, expensive and require experienced clinical staff. The SENATOR software has the capability of highlighting the most common potentially inappropriate prescriptions to a non-specialist clinician in an efficient manner and in so doing prompt alterations which will reduce risks of incident ADRs. If successful this will result in older patients suffering fewer adverse medication-related outcomes with associated economic benefits.

Prior to finalizing the trial protocol we conducted a non-interventional feasibility study at the same sites that are participating in the trial [23]. The feasibility study highlighted several challenges that led us to modify our initial trial design and our approach to the assessment of ADRs in the current trial.

We had initially proposed a cluster randomized trial adjusting for difference in baseline ADR risk using an ADR prediction tool such as the previously validated Gerontonet ADR Risk Scale [6]. This approach had the specific advantage of limiting contamination between the intervention and control arm, especially if it transpired that the same investigator was simultaneously attending a subject in both arms of the trial. However, in our feasibility study we discovered a very large degree of heterogeneity in ADR rates between different sites and between specialities within individual sites. Some of this heterogeneity may have resulted from initial limited standardization of our ADR reporting and adjudication processes and from sampling variability given the limited sizes of individual samples as well as real substantive differences between sites. Furthermore, we found that within our population the ADR predictions tools were inadequate for correcting for the between-cluster variability in baseline ADR risk. This made it impossible to exclude the possibility that any observed differences in the proposed trial might not simply be the consequence of an unequal distribution of baseline ADR risks across sites. We therefore adopted an individual level randomization, accepting that a degree of cross arm contamination might dilute the perceived effect size. However, even in a cluster design this effect is not fully prevented because junior medical staff routinely migrate between various specialities within a hospital and senior clinicians typically cross cover other specialist services at weekends and when working on-call outside of regular daytime hours.

The assessment of ADRs gives rise to several methodological complexities that have not previously been widely discussed in the literature. It is necessary to distinguish between prevalent (pre-dating the hospitalization and on occasion necessitating it) and incident ADRs, which occur during the index hospital stay. These are likely to differ in their characteristics, risk factors and consequences, making the distinction particularly relevant for ADR risk prediction tools. From the SENATOR trial viewpoint, it is necessary to exclude ADRs occurring prior to randomization; therefore outcomes in this trial will only include in-hospital incident ADRs that occur post randomization, but we also take account of whether identification and response to prevalent ADRs is influenced by study intervention.

The feasibility study highlighted the difficulty in reliably separating prolonged or repeated occurrences of the same ADR into separate discrete events in a reproducible fashion. This would necessitate defining a minimal interval between events and requires more detailed information on the timing of events than is usually present in clinical records or at least amenable to retrospective assessment. We therefore conceptualized ADRs as processes which are predisposed by one or more medications. This uses a patient-specific rather than a medication-specific perspective, therefore an ADR with two different potential implicated medications at different times is still considered a single potential endpoint. This potential loss of precision in defining ADRs is necessary for reproducibility and avoidance of an unduly subjective determination of the number of discrete episodes.

We adopted a retrospective approach to ADR assessment for two reasons. Firstly, real time assessment could potentially influence attending clinician or patient behaviour independently of the intervention. Secondly, there is the need to avoid situations where the site researcher identifies a potentially dangerous inappropriate prescribing in real time, leading to an ethical dilemma of whether or not to alert the attending clinical staff, thereby potentially distorting trial data. While a retrospective assessment may result in some ADRs not being clearly identified, our contention is that an adverse event that does not leave any evidence in the medical notes, nursing notes or elsewhere in the case records in the highly monitored hospital environment is more likely to be trivial in nature.

In keeping with the open label design that is unavoidable in a trial of a decision support tool and in view of the somewhat subjective nature of ADR ascertainment, it is necessary to use a detailed process to allow for blinded potential end-point adjudication. We optimized the function of this process by the use of an algorithm that varies the number of required potential end-point reviews depending on the degree of concordance between blinded reviewers and the strength of the initial reviewers’ assessment. We have also considered carefully the definitions used in our outcomes. Thus, the distinction between ‘certain’ and ‘probable’ ADR is less relevant as both are primary endpoints in contrast to the distinction between ‘possible’ and ‘probable’ ADR and between ‘possible’ and ‘unlikely’ ADR which have more influence on the secondary outcomes.

SENATOR is a complex clinical trial with many challenges. For this trial to succeed, attending clinicians in the intervention arm need to implement the recommendations of the SENATOR report. To date, adherence with software-generated prescribing advice in other studies has been highly variable [24]. In addition, ADRs need to be recorded and adjudicated in a comprehensive and unbiased fashion. SENATOR is the first multi-national randomized trial in which a prescribing optimization software specially designed to minimize ADRs and potential prescribing omissions will be tested on a large scale. If the SENATOR trial shows the software to be effective for minimizing ADRs and their consequences, there is the potential for routine prescribing optimization for large numbers of multi-morbid older people that is software-driven. A critical question that this trial will address is the extent to which the prescribing advice provided in the software-generated SENATOR report will be considered appropriate by busy clinicians and applied accordingly to their patients’ prescriptions in the acute hospital setting.

## Abbreviations

ADR: Adverse Drug Reaction
ONTOP: Optimal evidence-based Non-drug Therapies in Older People
SENATOR: Software ENgine for the Assessment and optimisation of drug and non-drug Therapy in Older peRsons
START: Screening Tool to Alert doctors to the Right Treatment
STOPP: Screening Tool of Older Persons’ Prescriptions
